# The opposite effect of ELP4 and ZEB2 on TCF7L2‐mediated microglia polarization in ischemic stroke

**DOI:** 10.1002/ccs3.12061

**Published:** 2025-01-16

**Authors:** Xiao‐li Min, Sixian Lin, Jia‐yi Hu, Rui Jing, Qing Zhao, Fei‐fei Shang, Yong Zeng

**Affiliations:** ^1^ Department of Cerebrovascular Diseases The Second Affiliated Hospital of Kunming Medical University Kunming China; ^2^ Institute of Life Science Chongqing Medical University Chongqing China; ^3^ Department of Psychiatry The Second Affiliated Hospital of Kunming Medical University Kunming China

**Keywords:** ELP4, ischemic stroke, microglia M1 polarization, TCF7L2, ZEB2

## Abstract

Microglia M1 polarization plays important role in the development of ischemic stroke (IS). This study explored the role of transcription factor 7 like 2 (TCF7L2) in regulating microglia M1 polarization during IS. TTC staining was used to determine the cerebral infarction, and Nissl staining was applied to examine neuronal injury. The secretion levels of cytokines were measured using ELISA. The interaction between Zinc finger E‐Box binding homeobox 2 (ZEB2) and TCF7L2 was analyzed by Co‐IP, and H3K27ac enrichment in the TCF7L2 promoter was detected by ChIP assay. TCF7L2 knockdown reduced MCAO/R‐induced mice cerebral injury. TCF7L2 silencing or TAK‐242 (TLR4 antagonist) injection inhibited OGD/R‐induced microglia M1 polarization by repressing the TLR4/NF‐κB signal, and TCF7L2 knockdown combined with TAK‐242 treatment further inhibited microglia M1 polarization. TCF7L2 promoted transcriptional activation of TLR4. ELP4 enhanced H3K27ac‐mediated transcriptional activation of TCF7L2, and ZEB2 promoted the K48‐linked ubiquitination of TCF7L2. TCF7L2 overexpression abolished the inhibitory effect of ELP4 knockdown or ZEB2 overexpression on OGD/R‐induced microglia M1 polarization. TCF7L2 exacerbated cerebral injury by promoting microglia M1 polarization during IS progression. Mechanistically, ELP4 promoted TCF7L2 expression by promoting H3K27ac enrichment in the TCF7L2 promoter, while ZEB2 promoted TCF7L2 ubiquitination degradation.

## Graphical abstract

During IS progression, the upregulated ELP4 promoted the transcriptional activity of TCF7L2 by recruiting H3K27ac to the TCF7L2 promoter, and the decreased ZEB2 protected TCF7L2 protein from ubiquitination degradation. Therefore, the increased TCF7L2 exacerbated cerebral injury by promoting microglia M1 polarization.

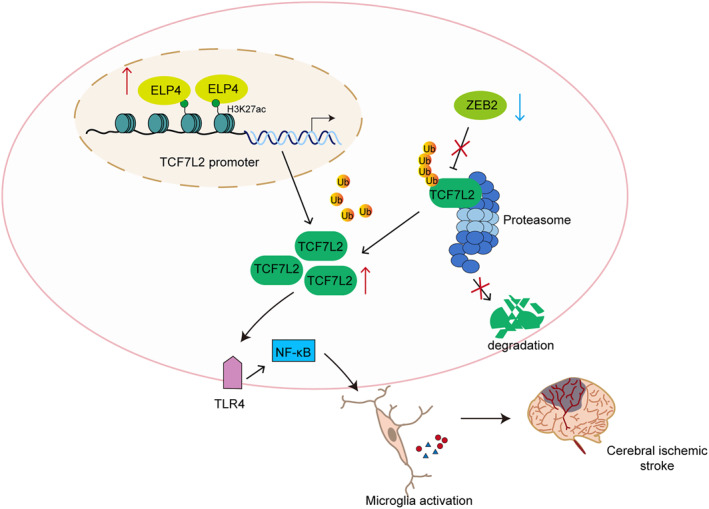

## 1 INTRODUCTION

Ischemic stroke (IS) is a major cause of death and disability worldwide.^1^ According to reports, IS kills around 5.5 million people globally each year, and an estimated 116.4 million people become permanently disabled due to IS.^2^ Currently, the only effective therapy for IS is intravenous thrombolysis^3^, ^4^; however, the current therapeutic options are limited due to the short treatment window.^4^ Therefore, novel IS treatment options must be developed as soon as possible. Microglia are brain‐resident macrophages capable of monitoring the microenvironment and initiate immune responses.^5^ Activated microglia are polarized into pro‐inflammatory M1 or anti‐inflammatory M2 phenotypes in response to diverse brain traumas, such as IS.^6^ M1 microglia facilitate secondary brain injury, while M2 microglia enhance recovery following IS.^7^ Therefore, inhibiting M1 polarization of microglia is a promising treatment option targeting IS, and elucidating the mechanisms involved is expected to provide profound research ideas for the treatment of IS.

Transcription factor 7 like 2 (TCF7L2) is a transcription factor with highly conserved sequence regions that is an essential component of the Wnt signaling pathway.^8^ The role of TCF7L2 in regulating IS progression has been studied. As evidence, chemokine (C‐X‐C motif) receptor 7 relieved IS by inhibiting TCF7L2 expression.^9^ Notably, a previous study showed that TCF7L2 upregulation promoted microglia activation by activating inflammation related signal in neuropathic pain.^10^ However, whether TCF7L2 promotes IS progression by facilitating microglia M1 polarization remains unclear and warrants more investigation.

The Elongator acetyltransferase complex subunit 4 (ELP4) protein is involved in transcriptional elongation, ribonucleic acid modification transfer, and polarized exocytosis.^11^ ELP4 gene mutations and deletions have been linked to a variety of neurological diseases.^12^, ^13^ However, its role in IS remains uncertain. Our previous research study found that ELP4 expression was markedly elevated in the peripheral blood of acute IS patients, indicating that ELP4 may play a role in IS development. As reported, ELP4 is essential for the activity of histone acetylase (HAT) and may promote histone acetylation levels of downstream gene promoters by synergistically interacting with histone acetylases (such as P300) to facilitate gene transcription.^14^ In the current study, it was predicted that acetylation of lysine 27 on histone 3 (H3K27ac) modification was enriched in TCF7L2 promoter using the UCSC database. Therefore, it's speculated that ELP4 regulates microglial polarization during IS progression by enhancing H3K27ac enrichment in the TCF7L2 promoter and promoting transcriptional activation of TCF7L2.

Zinc finger E‐Box binding homeobox 2 (ZEB2) encodes a zinc finger homologous domain transcription factor protein that regulates wound healing, development, cell migration, cell adhesion, and cytoskeleton reorganization.^15^, ^16^ The function of ZEB2 in central nervous system (CNS) under pathological conditions has been reported. For example, Vivinetto et al. demonstrated that conditional ZEB2 knockdown in astrocytes reduced astrogliosis, caused bigger lesions, and delayed recovery of motor function after CNS injury.^17^ More importantly, ZEB2‐enriched exosomes induced endogenous neurogenesis and improved functional recovery following IS.^18^ Herein, we found through bioinformatics prediction that ZEB2 may be a ubiquitinase that regulates the stability of TCF7L2 protein. As a result, it's speculated that ZEB2 may regulate microglial polarization during IS development by regulating the stability of TCF7L2 protein.

Based on the above evidence, we assumed that TCF7L2 upregulation promotes microglia M1 polarization and exacerbates neuronal injury during IS progression. Mechanistically, ELP4 promotes the activation of TCF7L2 transcription, while ZEB2 downregulation loses its ubiquitination degradation effect on TCF7L2 protein. Our research study reveals that TCF7L2 may be an important target for regulating microglia M1 polarization during IS progression.

## 2 MATERIALS AND METHODS

### 2.1 Cell culture and treatment

The human microglia (HMC3 cells) were purchased from ATCC (VA, USA). All cells were cultured in DMEM (Gibco, MD, USA) containing 10% FBS (Gibco) with 5% CO_2_ at 37°C. For the oxygen–glucose deprivation/reoxygenation (OGD/R) treatment, HMC3 cells were cultured in glucosefree DMEM (Gibco) under hypoxic conditions (95% N_2_ and 5% CO_2_) for 2 h, after which reoxygenation occurred (2% O_2_ and 5% CO_2_) for 24 h. For protein synthesis inhibition, cells were treated with 100 μg/mL cycloheximide (CHX, protein synthesis inhibitor, MedChemExpress, NJ, USA) for 0, 3, 6, and 9 h. For proteasome inhibition, cells were subjected to 5 μM MG132 (proteasome inhibitor, Sigma‐Aldrich, MO, USA) for 12 h.

### 2.2 Animal experiments

Male C57BL/6J mice (7‐8 week old, 20–25 g) were obtained from Charies River (Beijing, China). Mice were randomly divided into six groups: sham, middle cerebral artery occlusion/reperfusion (MCAO/R), MCAO/*R* + sh‐NC, MCAO/*R* + sh‐TCF7L2, MCAO/*R* + TAK‐242, and MCAO/*R* + sh‐TCF7L2 + TAK‐242 groups. Mice were anesthetized by pentobarbital sodium intraperitoneal injection (Sino Pharm, Shanghai, China) and submitted to MCAO or sham operation.^19^ Briefly, a monofilament nylon suture was inserted into the right internal carotid artery via the external carotid artery until mild resistance was encountered. The suture was removed after 90 min of MCAO. GenePharma (Shanghai, China) provided sh‐TCF7L2 and sh‐NC lentivirus. Lentivirus (5 μL, titer 2 × 10^8^ U/mL) were injected into brains (same side of MCAO/R) of mice in the MCAO/*R* + sh‐NC, MCAO/*R* + sh‐TCF7L2, and MCAO/*R* + sh‐TCF7L2 + TAK‐242 groups 24 h before surgery. The dissolved TAK‐242 (also named as resatorvid, 1 μΜ, ApexBio, TX, USA, A3850) or DMSO (1%) was injected intraperitoneally (3 mg/kg body weight) 1 h after MACO or sham operation as previously described.^20^ 48 h after MCAO, mice were sacrificed, and the brain tissue was collected. Animal experiments were conducted in four sections, which were used for TTC + neurological function analysis, brain water content, pathological detection, and biochemical detection, respectively. The number of animals in per group/each section was five. The animal studies were approved by Ethical Review Committee of Animal Experiments in Kunming Medical University (Ethics Approval No. kmmu20220412).

### 2.3 Measurement of dry‐wet weight ratio

After decapitation, the cerebral cortex, basal nucleus, and cerebellum were immediately weighed and baked to constant weight in a 60°C electric oven. The dried brains were then weighed swiftly. The dry–wet weight ratio was determined as (wet weight—dry weight)/wet weight × 100%.

### 2.4 Neurobehavior assessment

Neurological deficits were evaluated using the previously mentioned ^21^ modified neurologic severity score (mNSS). The mNSS was scored on a scale of 0–18 (18, greatest neurological deficit; 13–18, severe impairment; 7–12, moderate impairment; one to six mild impairment; 0, normal neurobehavioral score).

### 2.5 2,3,5‐Triphenyltetrazolium chloride (TTC) staining

The brain sections (4 μm in thickness) were prepared 48 h after MCAO and stained with 2% TTC (Sigma‐Aldrich). The sections were observed under an Olympus microscope (Tokyo, Japan). Five serial brain tissue slices were imaged, and ImageJ software was applied to analyze the infarct area of each slice. Infarct area (%) = (contralateral area—ipsilateral non‐infarct area)/contralateral area × 100%.

### 2.6 Nissl staining

The brain tissues were fixed in 10% paraformaldehyde and cut into 4 μm thick slices. The sections were incubated overnight with anhydrous ethanol and chloroform before being dehydrated. The sections were then stained with 0.1% methylphenol (Sigma‐Aldrich) for 10 min and observed under an Olympus inverted microscope.

### 2.7 Immunofluorescence staining

After dewaxing and antigen repair, the brain tissue sections were incubated with 3% hydrogen peroxide solution for 25 min. The sections were then incubated overnight with antibodies against Iba‐1 (Abcam, Cambridge, UK, 1:200, ab178846) and TCF7L2 (Proteintech, IL, USA, 1:200, 13838‐1‐AP) followed incubation with the corresponding secondary antibody (Abcam, 1:300, ab150077) for 1 h. The sections were sealed with the sealing liquid and observed under an Olympus fluorescence microscope.

### 2.8 Cell transfection

GenePharma (Shanghai, China) provided the short hairpin RNAs (sh‐TCF7L2 and sh‐ELP4), the overexpression plasmid (oe‐TLR4, oe‐TCF7L2, and oe‐ZEB2) and their negative controls. The vectors and shRNAs were introduced into cells using Lipofectamine 3000 (Invitrogen, CA, USA, L3000015).

### 2.9 Real‐time quantitative polymerase chain reaction (RT‐qPCR)

The total RNA was extracted from tissues and cells using TRIzol (Invitrogen). The reverse transcription kit (ThermoFisher Scientific, MA, USA) was used to reverse transcribe RNA samples. RT‐qPCR was performed using SYBR (ThermoFisher Scientific). GAPDH was used as the reference gene. The data were analyzed using the 2^−ΔΔCT^ method. The primers used in the study were listed as follows (5′‐3′):

CD16 (human) (F): ATCTTCAAGCAGGGAAGCCC.

CD16 (human) (R): TGTTGCTTTGCTGTGAGGGA.

CD16 (mouse) (F): CAGAATGCACACTCTGGAAGC.

CD16 (mouse) (R): GGGTCCCTTCGCACATCAG.

CD32 (human) (F): AGCCAATCCCACTAATCCTGA.

CD32 (human) (R): GGTGCATGAGAAGTGAATAGGTG.

CD32 (mouse) (F): GGAATCCTGCCGTTCCTACTG.

CD32 (mouse) (R): ATGGCACAAAGTCCGTGAGAA.

TLR4 (human) (F): AGACCTGTCCCTGAACCCTAT.

TLR4 (human) (R): CGATGGACTTCTAAACCAGCCA.

iNOS (human) (F): TTCAGTATCACAACCTCAGCAAG.

iNOS (human) (R): TGGACCTGCAAGTTAAAATCCC.

iNOS (mouse) (F): GGAGTGACGGCAAACATGACT.

iNOS (mouse) (R): TCGATGCACAACTGGGTGAAC.

CD206 (human) (F): TCCGGGTGCTGTTCTCCTA.

CD206 (human) (R): CCAGTCTGTTTTTGATGGCACT.

CD206 (mouse) (F): CTCTGTTCAGCTATTGGACGC.

CD206 (mouse) (R): TGGCACTCCCAAACATAATTTGA.

Arg1 (human) (F): GTGGAAACTTGCATGGACAAC.

Arg1 (human) (R): AATCCTGGCACATCGGGAATC.

Arg1 (mouse) (F): CTCCAAGCCAAAGTCCTTAGAG.

Arg1 (mouse) (R): GGAGCTGTCATTAGGGACATCA.

CD163 (human) (F): TTTGTCAACTTGAGTCCCTTCAC.

CD163 (human) (R): TCCCGCTACACTTGTTTTCAC.

CD163 (mouse) (F): GGTGGACACAGAATGGTTCTTC.

CD163 (mouse) (R): CCAGGAGCGTTAGTGACAGC.

TCF7L2 (human) (F): TGGAGGGCTCTTTAAGGGG.

TCF7L2 (human) (R): GATCCGTTGGGGAGGTAGG.

TCF7L2 (mouse) (F): TCATCACGTACAGCAATGAACA.

TCF7L2 (mouse) (R): CGACAGCGGGTAATATGGAGAG.

ELP4 (human) (F): AAGAGCAACGTCACCAGTTTC.

ELP4 (human) (R): GGAGCCCGGTTGATACCAG.

ZEB2 (human) (F): CAAGAGGCGCAAACAAGCC.

ZEB2 (human) (R): GGTTGGCAATACCGTCATCC.

β‐actin (human) (F): TGGCACCACACCTTCTACAA.

β‐actin (human) (R): CCAGAGGCGTACAGGGATAG.

β‐actin (mouse) (F): GTGACGTTGACATCCGTAAAGA.

β‐actin (mouse) (R): GCCGGACTCATCGTACTCC.

### 2.10 Western blot analysis

The proteins were extracted from cells and tissues by RIPA buffer (Beyotime). After assessing the concentration of proteins using the BCA kit (ThermoFisher Scientific), the protein samples were isolated by 10% SDS‐PAGE and transferred to a Millipore PVDF membrane. The membranes were blocked and incubated overnight with TCF7L2 (ab272235), TLR4 (ab13556), p‐NF‐κB p65 (ab239882), NF‐κB p65 (ab207297), ELP4 (ab133687), ZEB2 (ab138222), and *β*‐actin (ab8227). The membranes were then hybridized with the secondary antibody (ab7090) for 60 min. The protein bands were visualized by ECL and quantified by Image J. All antibodies were purchased from Abcam and diluted according to the instructions.

### 2.11 Enzyme linked immunosorbent assay (ELISA)

The levels of tumor necrosis factor (TNF)‐α, interleukin (IL)‐1β and IL‐6 were detected by the human/mouse TNF‐α ELISA kit (Abcam, ab181421/ab208348), the human/mouse IL‐1β ELISA kit (Abcam, ab214025/ab197742) and the human/mouse IL‐6 ELISA kit (Abcam, ab178013/ab222503), respectively. All operations were strictly carried out in accordance with the instructions. The OD values were recorded at 450 nm and analyzed by Origin 9.5 software.

### 2.12 Flow cytometry

Cell concentration was diluted to 1 × 10^7^/ml using PBS containing 10% FBS. Cells were then stained for 30 min with anti‐CD86 (Abcam, 1:500, ab239075) in dark. Following two washes, cells were suspended in 2 ml PBS and analyzed by flow cytometry (BD, NJ, USA).

### 2.13 Chromatin immunoprecipitation (ChIP) assay

Cells were fixed with 1% formaldehyde for 5 min to induce DNA–protein cross‐linking. Cell lysate was then ultrasonically treated to produce chromatin fragments, and incubated with anti‐H3K27ac (Abcam, 1:60, ab4729), anti‐TCF7L2 (Sigma‐Aldrich, 1:50, 05–511), or anti‐IgG (Abcam, 1:100, ab172730). Pierce protein A/*G* beads (ThermoFisher Scientific) was used to isolate chromatin‐antibody complexes. DNA was purified and analyzed using RT‐qPCR.

### 2.14 Analysis of TCF7L2 ubiquitination

To detect ubiquitination levels of TCF7L2, HA‐K48‐Ub plasmids were transfected into HMC3 cells. Then cell lysates were immunoprecipitated with IgG (Abcam, 1:100, ab172730) or TCF7L2 antibody (Proteintech, 1:50, 13838‐1‐AP) and incubated for 12 h with protein A/*G* IP magnetic beads. Proteins were resolved by SDS‐PAGE and immunoblotted with anti‐HA (Abcam, 1:1000, ab236632) antibody.

### 2.15 Coimmunoprecipitation (Co‐IP)

Cells were lysed in lysis solution. The Sepharose CL‐4B beads (Sigma‐Aldrich) were incubated at 4°C with the primary antibodies against IgG (Abcam, 1:50, ab172730) or ZEB2 (Proteintech, 1:50, 14026‐1‐AP) for 4 h. The beads were then incubated with cell lysate overnight. The protein bound were then eluted and analyzed using Western blot.

### 2.16 Data analysis

All our data were obtained from three independent experiments. The statistical data analyzed by Graphpad Prism 7.0 and expressed as means ± SD. The differences between the two groups were investigated using Student's *t*‐tests. One‐way ANOVA followed by Tukey's post hoc test was performed to compare differences between groups. The *p*‐values less than 0.05 were regarded as significant.

## 3 RESULTS

### 3.1 TCF7L2 knockdown inhibited MCAO/R‐induced cerebral injury in mice

**FIGURE 1:**
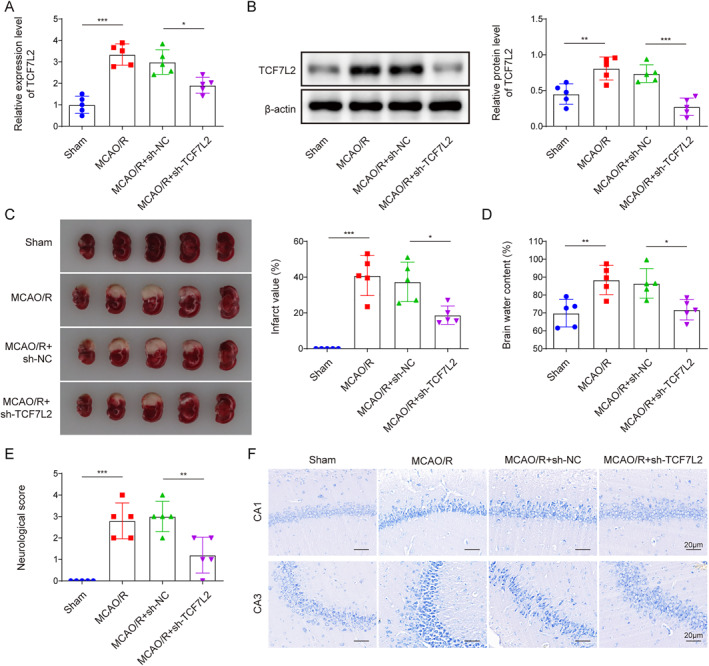
TCF7L2 knockdown inhibited MCAO/R‐induced cerebral injury in mice. Mice were subjected to MCAO/R treatment, and mice were injected with the lentivirus packaging with sh‐TCF7L2 or sh‐NC at 24 h before‐surgery. (A‐B) The mRNA and protein levels of TCF7L2 in brain tissues were determined by RT‐qPCR and western blot, respectively. (C) TTC staining was employed to detect cerebral infarct size. (D) The dry‐wet weight ratio of brain tissues was measured. (E) mNSS was performed to assess neurological deficits. (F) The representative images of Nissl staining of brain tissues were presented, and the relevant indicators were quantified. Data were expressed as mean ± SD. *p < 0.05, **p < 0.01, ***p < 0.001.

To investigate the role of TCF7L2 in IS, we employed an ischemic stroke model (MCAO/R mice), and lentivirus‐packed sh‐TCF7L2 and sh‐NC were injected into MCAO/R mice brain at 24 h before‐surgery. TCF7L2 expression level in brain tissues was markedly elevated by MCAO/R treatment, while this change was reversed by TCF7L2 knockdown (Figure 1A–B). TCF7L2 knockdown also reduced MCAO‐induced cerebral infarct size (Figure 1C). In addition, MCAO/R caused brain edema in mice, which was alleviated by TCF7L2 silencing (Figure 1D). Moreover, TCF7L2 knockdown reduced MCAO/R‐induced neurological deficits in mice (Figure 1E). It also turned out that MCAO/R caused neuronal injury in the hippocampal CA1 and CA3 regions of mouse brain tissues, which was improved by TCF7L2 knockdown (Figure 1F). Collectively, TCF7L2 expression was increased in brain tissues of MCAO/R mice, and its silencing mitigated cerebral injury.

### 3.2 TCF7L2 knockdown reduced MCAO/R‐induced inflammatory response in brain tissues by inactivating the TLR4/NF‐κB signal

**FIGURE 2:**
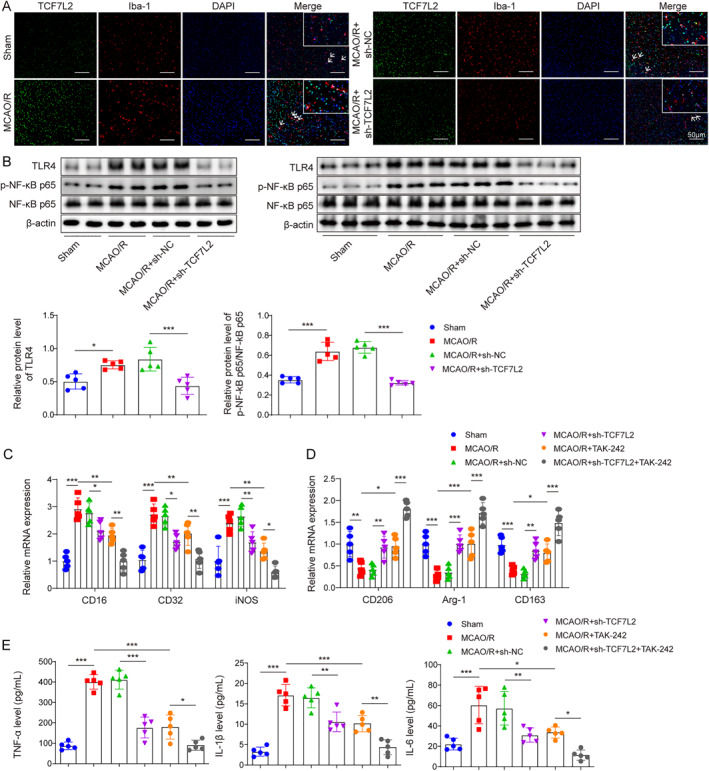
TCF7L2 knockdown reduced MCAO/R‐induced inflammatory response in brain tissues by inactivating the TLR4/NF‐κB signal. Mice were subjected to MCAO/R treatment, and mice were injected with the lentivirus packaging with sh‐TCF7L2 or sh‐NC at 24 h before‐surgery. A, The co‐expression of Iba‐1 (microglial marker) and TCF7L2 in the cerebral cortex was analyzed by immunofluorescence staining. B, Western blot was employed to detect TLR4, p‐NF‐κB p65 and NF‐κB p65 protein levels in brain tissues. The dissolved TAK‐242 or DMSO (1%) was also injected intraperitoneally (3 mg/kg body weight) 1 h after MACO or sham operation. C–D, The mRNA levels of CD16, CD32, iNOS, CD206, Arg‐1, and CD163 were measured by RT‐qPCR. E, ELISA was performed to determine TNF‐α, IL‐1β and IL‐6 levels in brain tissues. Data were expressed as mean ± SD. *p < 0.05, **p < 0.01, ***p < 0.001.

It was initially shown that MCAO/R enhanced the co‐expression of Iba‐1 (microglial marker) and TCF7L2 in the cerebral cortex, whereas this change was partially eliminated by TCF7L2 knockdown (Figure 2A). The TLR4/NF‐κB signal is a classic pro‐inflammatory signaling pathway.^22^ Herein, it was discovered that TLR4 and p‐NF‐κB p65 protein levels in brain tissues were significantly increased by MCAO/R stimulation, while these protein expression changes were partially reversed by TCF7L2 knockdown (Figure 2B). As reported, the intra‐peritoneal injection of TAK‐242 (an antagonist for TLR4) markedly reduced cerebral infarction and improved neurologic function in mice with acute cerebral ischemia/reperfusion injury.^20^ In addition, TLR4 inhibition could attenuate microglia inflammatory response.^23^ Our results showed that MCAO/R treatment markedly elevated the expressions of M1 markers (CD16, CD32, and iNOS) and reduced the expressions of M2 markers (CD206, Arg‐1, and CD163) in brain tissues, but these changes were eliminated by TCF7L2 silencing or TAK‐242 injection, and knocking down TCF7L2 while administering intraperitoneal TAK‐242 injection could further suppress microglia M1 polarization (Figure 2C–D). Moreover, TNF‐α, IL‐1 *β* and IL‐6 levels in brain tissues were significantly increased following MCAO/R stimulation, while TCF7L2 knockdown or TAK‐242 injection weakened these changes, and injecting TAK‐242 intraperitoneally combined with silencing of TCF7L2 could further improved the inflammatory responses in brain tissue (Figure 2E). Taken together, TCF7L2 knockdown reduced MCAO/R‐induced inflammatory response in brain tissues by inhibiting microglia M1 polarization through inactivating the TLR4/NF‐κB signal.

### 3.3 TCF7L2 knockdown inhibited OGD/R‐induced microglia M1 polarization by inactivating the TLR4/NF‐κB signal

HMC3 cells were subjected to OGD/R treatment to establish an in vitro IS model. As demonstrated in Figure 3A–B, OGD/R treatment markedly elevated CD16, CD32 and iNOS expressions and reduced CD206, Arg‐1 and CD163 expressions in HMC3 cells. Meanwhile, TNF‐α, IL‐1β and IL‐6 secretion levels in HMC3 cells were significantly increased by OGD/R stimulation (Figure 3C). Notably, OGD/R markedly elevated TCF7L2 expression in HMC3 cells (Figure 3D–E). sh‐TCF7L2 or sh‐NC was transfected into HMC3 cells to knockdown TCF7L2. It turned out that sh‐TCF7L2 transfection markedly decreased TCF7L2 expression in HMC3 cells (Figure 3F–G). As shown in Figure 3H–I, sh‐TCF7L2 transfection ameliorated OGD/R‐induced increase in TCF7L2 expression in HMC3 cells. A recent study has found that TCF7L2 promoted TLR4 transcription by binding to the TLR4 promoter, thereby further regulating the TLR4/NF‐κB signaling.^10^ In the current study, the result from ChIP assay also confirmed the binding relationship between TCF7L2 and TLR4 promoter (Figure S1A). It was also observed that OGD/R treatment significantly elevated TLR4 mRNA level in HMC3 cells, while this upregulation was reversed by TCF7L2 knockdown (Figure S1B). In addition, TCF7L2 knockdown reversed the promoting effect of OGD/R on TLR4 and p‐NF‐κB p65 protein levels in HMC3 cells (Figure 3J). To further investigate the interaction between TCF7L2 and TLR4/NF‐κB signal in regulating microglia M1 polarization during IS progression, both TCF7L2 knockdown and TLR4 overexpression were induced in OGD/R‐treated HMC3 cells. The transfection efficiency of oe‐TLR4 was shown in Figure 3K–L, and the results showed that oe‐TLR4 transfection significantly increased the mRNA and protein levels of TLR4 in HMC3 cells, indicating that the transfection was successful. It was subsequently revealed that TCF7L2 knockdown ameliorated OGD/R‐induced increase in CD16, CD32, and iNOS expressions in HMC3 cells and prevented OGD/R‐induced decrease in CD206, Arg‐1, and CD163 expressions, whereas these effects of TCF7L2 knockdown were reversed by the co‐transfection of oe‐TLR4 (Figure 3M–N). Flow cytometry result also showed that mediated downregulation of M1 marker CD86 was abolished by TLR4 overexpression in OGD/R‐treated HMC3 cells (Figure 3O). Moreover, OGD/R‐mediated promotion on TNF‐α, IL‐1β, and IL‐6 secretion levels in HMC3 cells was ameliorated by TCF7L2 knockdown, which was further reversed by upregulation of TLR4 (Figure 3P). Taken together, TCF7L2 knockdown reduced OGD/R‐induced microglia M1 polarization by inactivating the TLR4/NF‐κB signal.

FIGURE 3TCF7L2 knockdown inhibited OGD/R‐induced microglia M1 polarization by inactivating the TLR4/NF‐κB signal. HMC3 cells were subjected to OGD/R treatment to construct an in vitro model of IS. (A‐B) The mRNA levels of CD16, CD32, iNOS, CD206, Arg‐1, and CD163 were examined by RT‐qPCR. (C) TNF‐α, IL‐1β and IL‐6 secretion levels in HMC3 cells were detected by ELISA. (D‐E) The mRNA and protein levels of TCF7L2 in HMC3 cells were determined by RT‐qPCR and western blot, respectively. (F‐G) The mRNA and protein levels of TCF7L2 in HMC3 cells after sh‐NC or sh‐TCF7L2 transfection were analyzed using RT‐qPCR and western blot, respectively. HMC3 cells were transfected with sh‐NC or sh‐TCF7L2 combined with OGD/R treatment. (H‐I) The mRNA and protein levels of TCF7L2 in HMC3 cells were examined using RT‐qPCR and western blot, respectively (J) TLR4, p‐NF‐κB p65 and NF‐κB p65 protein levels in cells were measured by western blot. (K‐L) The mRNA and protein levels of TLR4 in HMC3 cells after oe‐NC or oe‐TLR4 transfection were detected by RT‐qPCR and western blot. Both TCF7L2 knockdown and TLR4 overexpression were induced in OGD/R‐treated HMC3 cells. (M‐N) RT‐qPCR was adopted to detect CD16, CD32, iNOS, CD206, Arg‐1, and CD163 mRNA levels in cells. (O) Flow cytometry was performed to analyze CD86 level in cells. (P) ELISA was employed to determine TNF‐α, IL‐1β and IL‐6 secretion levels in cells. Data were expressed as mean ± SD. All our data were obtained from three independent experiments. *p < 0.05, **p < 0.01, ***p < 0.001.

**Figure:**
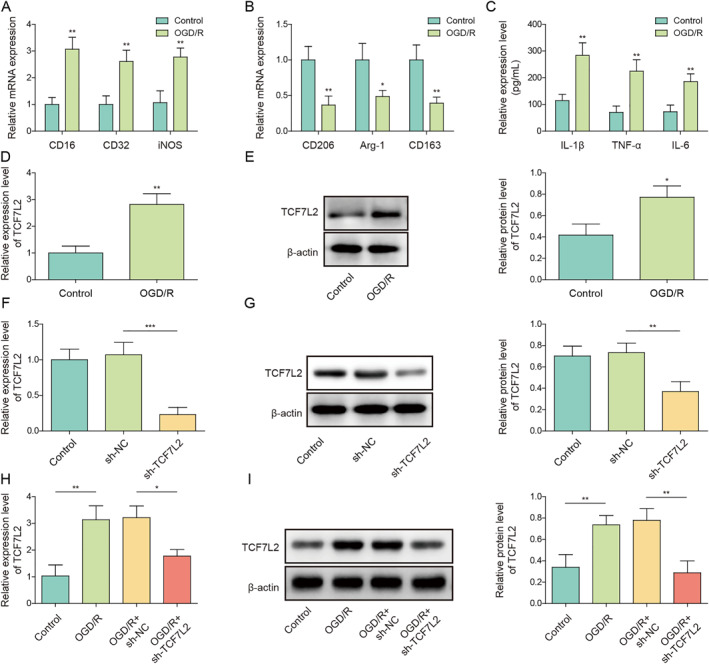

**Figure:**
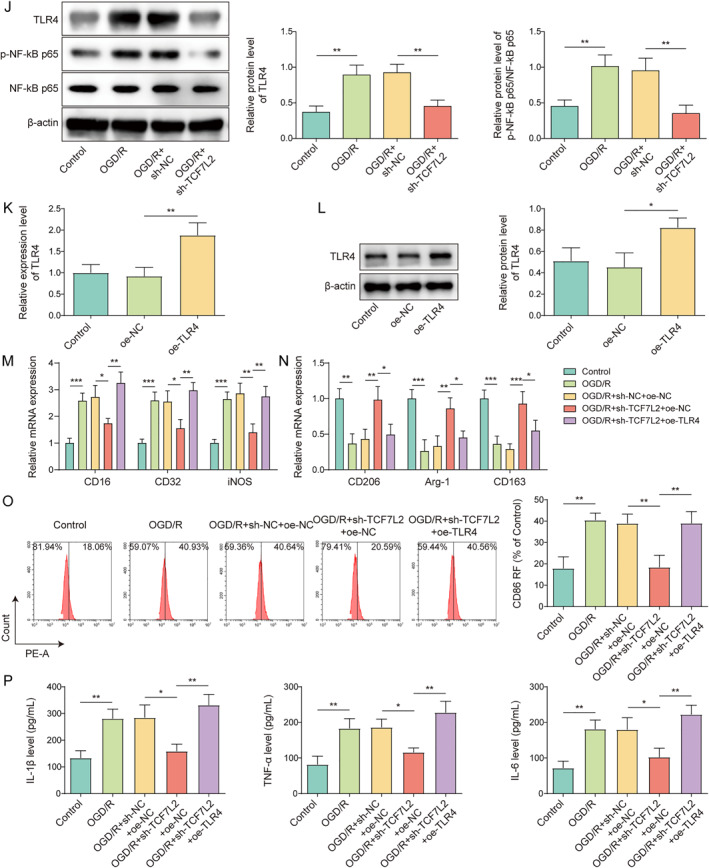

### 3.4 ELP4 promoted the enrichment of H3K27ac in the TCF7L2 promoter region in microglia

**FIGURE 4:**
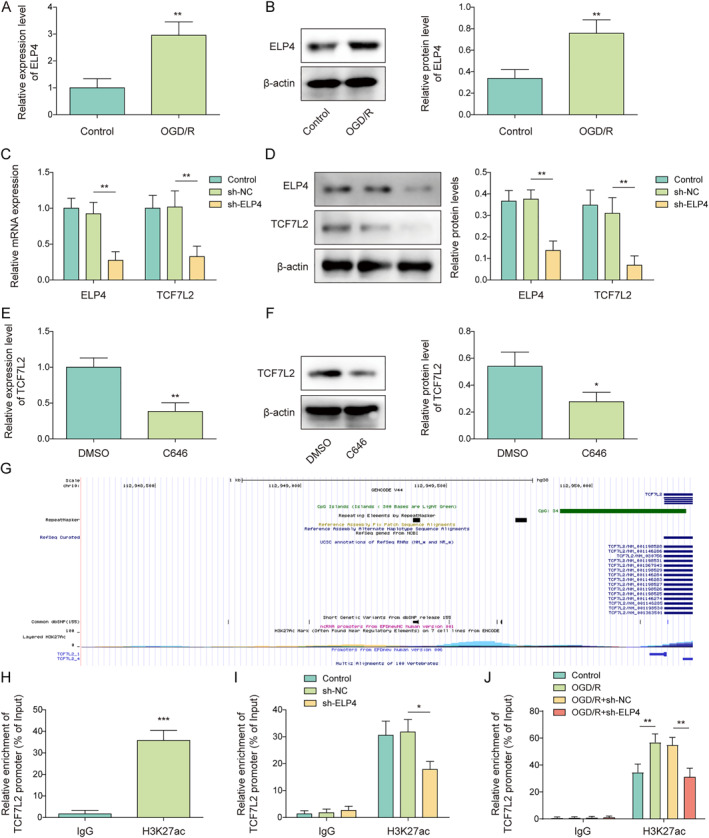
ELP4 promoted the enrichment of H3K27ac in the TCF7L2 promoter region in microglia. (A‐B) The mRNA and protein levels of ELP4 in HMC3 cells under OGD/R condition were determined by RT‐qPCR and western blot, respectively. (C‐D) The mRNA and protein levels of ELP4 and TCF7L2 in HMC3 cells after sh‐NC or sh‐ELP4 transfection were analyzed using RT‐qPCR and western blot, respectively. (E‐F) The mRNA and protein levels of TCF7L2 in HMC3 cells after DMSO or C646 treatment were analyzed using RT‐qPCR and western blot, respectively. (G) The UCSC (http://genome.ucsc.edu/) database was employed to analyze H3K27ac enrichment in the TCF7L2 promoter region. (H‐I) ChIP assay was adopted to analyze the interaction between ELP4 and TCF7L2. (J) OGD/R‐treated HMC3 cells were transfected with sh‐NC or sh‐ELP4, and the enrichment of H3K27ac in the TCF7L2 promoter region in cells was analyzed by ChIP assay. Data were expressed as mean ± SD. All our data were obtained from three independent experiments. *p < 0.05, **p < 0.01, ***p < 0.001.

ELP4 is a protein that promotes histone acetylation levels of downstream gene promoters by synergistically interacting with histone acetylases to facilitate gene transcription.^14^ Our results showed that OGD/R treatment significantly elevated the mRNA and protein levels of ELP4 in HMC3 cells (Figure 4A–B). We subsequently induced ELP4 knockdown in HMC3 cells. It was observed that sh‐ELP4 transfection markedly reduced ELP4 and TCF7L2 expression levels in HMC3 cells (Figure 4C–D). Notably, C646 (HAT inhibitor) treatment significantly reduced TCF7L2 mRNA and protein levels in HMC3 cells (Figure 4E–F), suggesting that TCF7L2 expression in microglia might be regulated by acetylation. As predicted by the UCSC database, it was found that H3K27ac could be enriched in the TCF7L2 promoter region (Figure 4G). As confirmed by ChIP assay, H3K27ac was enriched in the TCF7L2 promoter region, while this enrichment was weakened by ELP4 silencing (Figure 4H–I). It also turned out that OGD/R treatment significantly increased H3K27ac enrichment in the TCF7L2 promoter region, whereas this change was eliminated by ELP4 knockdown (Figure 4J). In summary, ELP4 enhanced H3K27ac enrichment in the TCF7L2 promoter region and promoted TCF7L2 expression in microglia.

### 3.5 TCF7L2 overexpression reversed the inhibitory effect of ELP4 knockdown on OGD/R‐induced microglia M1 polarization

To investigate the role of ELP4 in regulating microglial polarization during IS development, HMC3 cells were transfected with sh‐ELP4 or sh‐NC combined with OGD/R treatment. As revealed in Figures 5A–C, sh‐ELP4 transfection ameliorated OGD/R‐induced increase in TCF7L2 and ELP4 expressions in HMC3 cells. It was subsequently demonstrated that ELP4 knockdown reduced CD16, CD32, and iNOS expressions in OGD/R‐treated HMC3 cells while elevating CD206, Arg‐1, and CD163 expressions in cells (Figures 5D–E). Meanwhile, OGD/R‐induced increase in TNF‐α, IL‐1β, and IL‐6 secretion levels in HMC3 cells was ameliorated by ELP4 knockdown (Figure 5F). Subsequently, both TCF7L2 overexpression and ELP4 knockdown were induced in HMC3 cells combined with OGD/R treatment to further study the role of ELP4 and TCF7L2 in regulating microglia polarization. It is observed that oe‐TCF7L2 transfection significantly elevated TCF7L2 expression in HMC3 cells (Figures 5G–H). TCF7L2 overexpression also prevented sh‐ELP4‐induced decrease in TCF7L2 in OGD/R‐treated HMC3 cells (Figures 5I–J). The results from RT‐qPCR subsequently showed that the inhibitory effect of ELP4 knockdown on CD16, CD32, and iNOS expressions in OGD/R‐treated HMC3 cells and the promoting effect on CD206, Arg‐1, and CD163 expressions were all abolished by TCF7L2 overexpression (Figure 5K–L). Flow cytometry results also displayed that ELP4 knockdown mitigated OGD/R‐induced increase in CD86 level in HMC3 cells, whereas the effect of sh‐ELP4 was eliminated by the co‐transfection of oe‐TCF7L2 (Figure 5M). Furthermore, TCF7L2 overexpression abrogated the inhibitory effect of ELP4 knockdown on TNF‐α, IL‐1β, and IL‐6 secretion levels in OGD/R‐treated HMC3 cells (Figure 5N). In summary, ELP4 promoted OGD/R‐induced microglia M1 polarization by mediating TCF7L2.

FIGURE 5TCF7L2 overexpression reversed the inhibitory effect of ELP4 knockdown on OGD/R‐induced microglia M1 polarization. HMC3 cells were transfected with sh‐NC or sh‐ELP4 combined with OGD/R treatment. (A‐C) The mRNA and protein levels of ELP4 and TCF7L2 in HMC3 cells were analyzed using RT‐qPCR and western blot, respectively. (D‐E) CD16, CD32, iNOS, CD206, Arg‐1, and CD163 mRNA levels in cells were examined by RT‐qPCR. (F) ELISA was employed to determine TNF‐α, IL‐1β, and IL‐6 secretion levels in cells. (G‐H) The mRNA and protein levels of TCF7L2 in HMC3 cells after oe‐NC or oe‐TCF7L2 transfection were analyzed using RT‐qPCR and western blot, respectively. Both TCF7L2 overexpression and ELP4 knockdown were induced in OGD/R‐treated HMC3 cells. (I‐J) The mRNA and protein levels of TCF7L2 in HMC3 cells were detected by RT‐qPCR and western blot, respectively. (K‐L) The mRNA levels of CD16, CD32, iNOS, CD206, Arg‐1 and CD163 were analyzed using RT‐qPCR. (M) CD86 level in cells was examined by flow cytometry. (N) ELISA was employed to determine IL‐1β, TNF‐α and IL‐6 secretion levels in cells. Data were expressed as mean ± SD. All our data were obtained from three independent experiments. *p < 0.05, **p < 0.01, ***p < 0.001.

**Figure:**
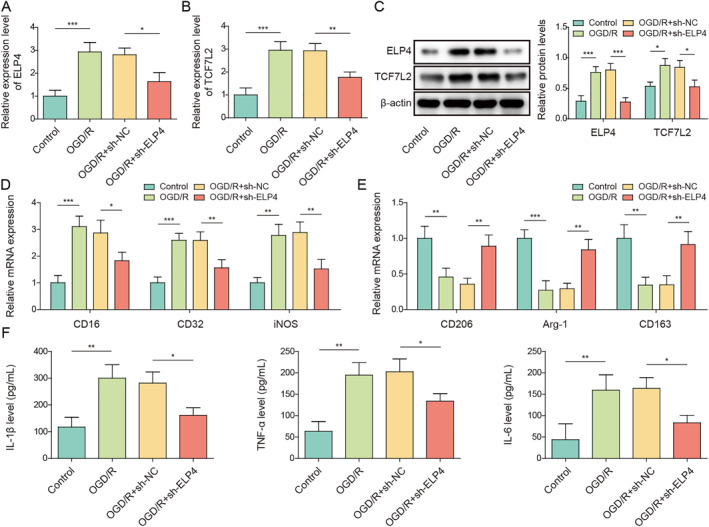

**Figure:**
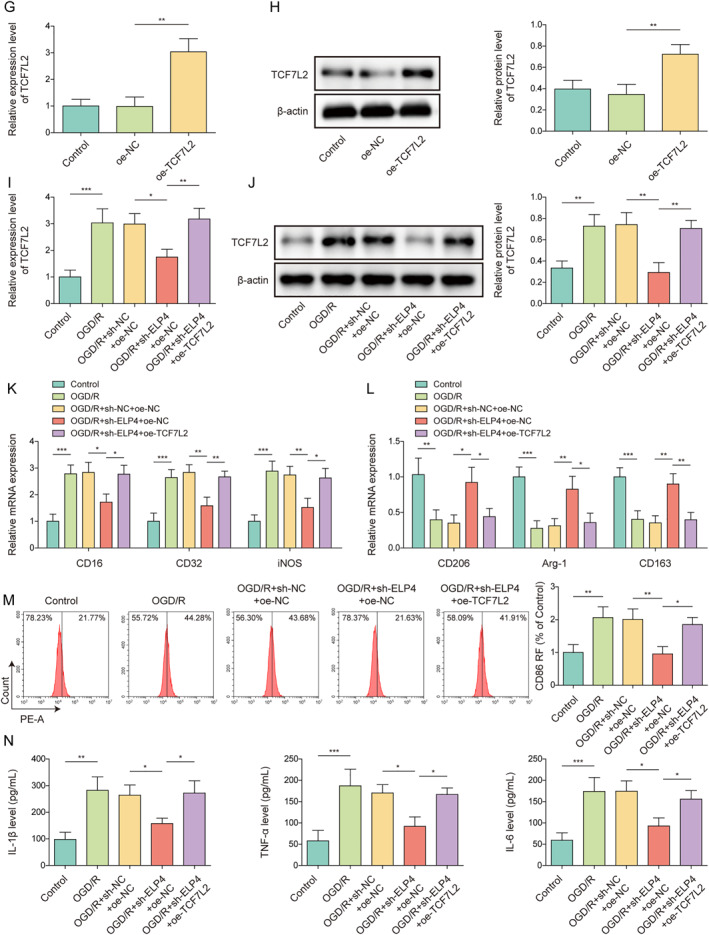

### 3.6 ZEB2 promoted the ubiquitination degradation of TCF7L2 protein by interacting with TCF7L2

The majority of biological activities in mammalian cells need the covalent modification of proteins with ubiquitin 24. Next, we further explored whether TCF7L2 is regulated by ubiquitination at the protein level. It was predicted that ZEB2 might be a ubiquitinase of TCF7L2 protein using the ubibrowser database (Figure 6A). As confirmed by Co‐IP assay, ZEB2 protein directly interacted with TCF7L2 protein (Figure 6B). It was found that, ZEB2 mRNA and protein levels in HMC3 cells were markedly reduced by OGD/R stimulation (Figure 6C–D). To detect the regulatory effect of ZEB2 on TCF7L2, overexpression was subsequently induced in HMC3 cells by transfecting oe‐ZEB2 into cells. It was discovered that oe‐ZEB2 transfection significantly increased ZEB2 expression in HMC3 cells (Figure 6E–F), indicating that the transfection was successful. Meanwhile, ZEB2 overexpression significantly reduced TCF7L2 protein level in HMC3 cells but did not affect TCF7L2 mRNA level (Figure 6G–H), suggesting that ZEB2 may regulate TCF7L2 protein level in microglia at the post transcriptional level. In addition, ZEB2 overexpression promoted TCF7L2 protein degradation in HMC3 cells under CHX (protein synthesis inhibitor) treatment condition (Figure 6I), suggesting that ZEB2 upregulation impaired TCF7L2 protein stability. Furthermore, MG132 (proteasome inhibitor) treatment reversed the inhibitory effect of ZEB2 overexpression on TCF7L2 protein level in HMC3 cells (Figure 6J), indicating that ZEB2 promoted the degradation of TCF7L2 by proteasomes. It has been reported that ubiquitin moieties linked to each other via lysine at position 48 (K48) represent the canonical signal for proteasomal degradation.^25^ Herein, it was found that OGD/R treatment markedly reduced the K48‐linked ubiquitination level of TCF7L2 in HMC3 cells (Figure 6K). As expected, ZEB2 overexpression promoted the formation of K48‐linked ubiquitination chain of TCF7L2 (Figure 6L). It also turned out that OGD/R treatment increased TCF7L2 expression and reduced ZEB2 expression in HMC3 cells, whereas these changes were eliminated after ZEB2 overexpression (Figure 6M–N). All these results indicated that ZEB2 catalyzed TCF7L2 degradation via K48‐linked ubiquitination.

FIGURE 6ZEB2 promoted the ubiquitination degradation of TCF7L2 protein by interacting with TCF7L2 (A) The ubibrowser database (http://ubibrowser.ncpsb.org/) was employed to predict the ubiquitinases acting on TCF7L2 protein. (B) The interaction between ZEB2 and TCF7L2 was analyzed by Co‐IP assay. (C–D) The mRNA and protein levels of ZEB2 in HMC3 cells under OGD/R condition were determined by RT‐qPCR and western blot, respectively. HMC3 cells were transfected with oe‐NC or oe‐ZEB2. (E–F) The mRNA and protein levels of ZEB2 in HMC3 cells were analyzed using RT‐qPCR and western blot, respectively. (G–H) The mRNA and protein levels of TCF7L2 in HMC3 cells were analyzed using RT‐qPCR and western blot, respectively. (I) CHX chase assay was employed to analyze TCF7L2 protein stability. (J) TCF7L2 protein level in cells after MG132 treatment was determined by western blot (K) The ubiquitination level of TCF7L2 in HMC3 cells after OGD/R treatment was analyzed by TCF7L2 ubiquitination analysis (L) TCF7L2 ubiquitination level in HMC3 cells after ZEB2 overexpression was analyzed by TCF7L2 ubiquitination analysis. HMC3 cells were transfected with oe‐NC or oe‐ZEB2 combined with OGD/R treatment (M) ZEB2 mRNA level in cells was detected using RT‐qPCR (N) Western blot was performed to assess TCF7L2 and ZEB2 protein levels in cells. Data were expressed as mean ± SD. All our data were obtained from three independent experiments. *p < 0.05, **p < 0.01, ***p < 0.001.

**Figure:**
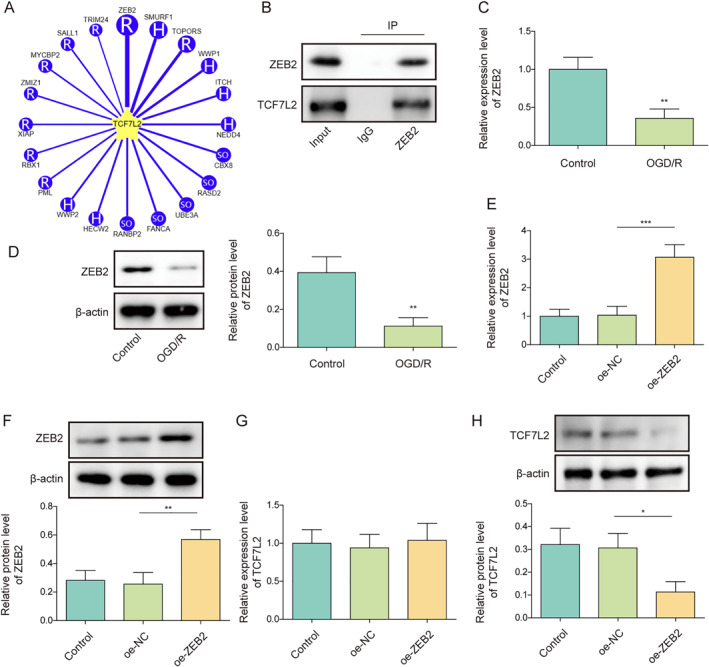

**Figure:**
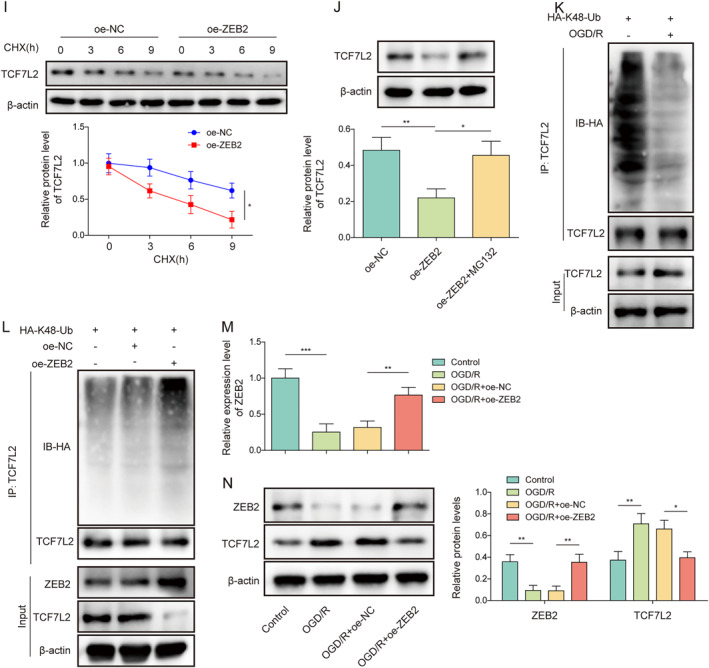

### 3.7 TCF7L2 overexpression reversed the inhibitory effect of ZEB2 overexpression on OGD/R‐induced microglia M1 polarization

**FIGURE 7:**
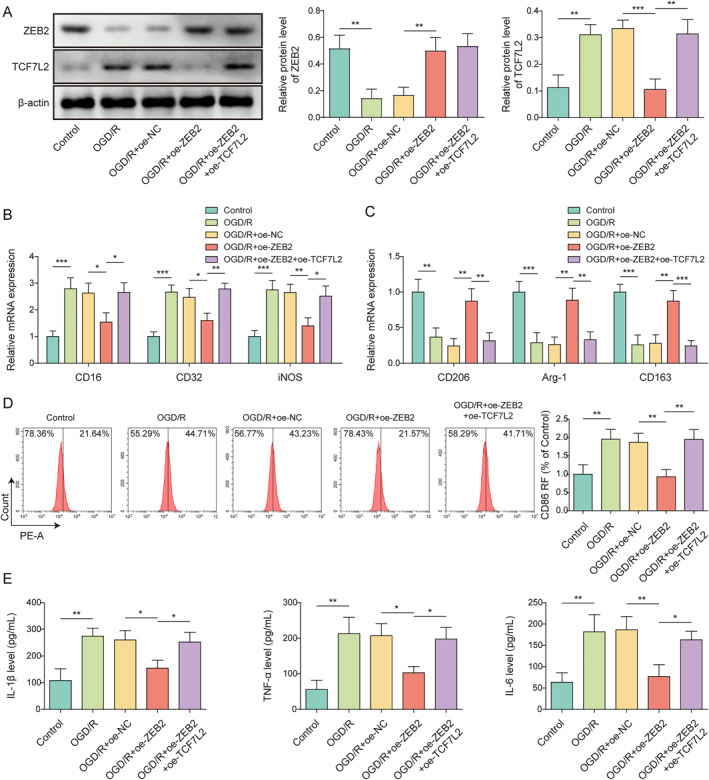
TCF7L2 overexpression reversed the inhibitory effect of ZEB2 overexpression on OGD/R‐induced microglia M1 polarization. Both TCF7L2 overexpression and ZEB2 overexpression were induced in HMC3 cells under OGD/R treatment. (A) TCF7L2 and ZEB2 protein levels in cells were assessed using western blot. (B‐C) RT‐qPCR was employed to determine CD16, CD32, iNOS, CD206, Arg‐1, and CD163 mRNA levels in cells. (D) Flow cytometry was performed to analyze CD86 level in cells. (E) TNF‐α, IL‐1β, and IL‐6 secretion levels in cells were determined using ELISA. Data were expressed as mean ± SD. All our data were obtained from three independent experiments. *p < 0.05, **p < 0.01, ***p < 0.001.

To study the interaction between ZEB2 and TCF7L2 in controlling microglia polarization, both TCF7L2 and ZEB2 upregulation were induced in HMC3 cells under OGD/R treatment. As shown in Figure 7A, OGD/R treatment significantly reduced ZEB2 protein levels and increased TCF7L2 protein level in HMC3 cells, whereas these changes induced by OGD/R were eliminated by oe‐ZEB2 transfection. It was also observed that the co‐transfection of oe‐TCF7L2 had no effect on ZEB2 protein level but reversed the inhibitory effect of ZEB2 overexpression on TCF7L2 protein level (Figure 7A). It was subsequently revealed that ZEB2 upregulation reversed the promoting effect of OGD/R on M1 markers (CD16, CD32, and iNOS) in HMC3 cells and the inhibitory effect on M2 markers (CD206, Arg‐1 and CD163), whereas these changes induced by ZEB2 overexpression were all eliminated by TCF7L2 overexpression (Figure 7B–C). Additionally, OGD/R‐induced increase in CD86 level in HMC3 cells was ameliorated by ZEB2 overexpression, while this change was abrogated by TCF7L2 upregulation (Figure 7D). Moreover, ZEB2 overexpression attenuated OGD/R‐induced increase in TNF‐α, IL‐1β and IL‐6 secretion levels in HMC3 cells, which was abolished by oe‐TCF7L2 co‐transfection (Figure 7E). In summary, ZEB2 overexpression abolished OGD/R‐induced microglia M1 polarization by regulating TCF7L2.

## 4 DISCUSSION

IS frequently causes irreversible brain injury and is a leading cause of mortality and morbidity worldwide.^26^ Despite substantial attempts to develop effective medicines, progress has been slow, and no innovative medications for brain protection are available.^4^ Therefore, there is an urgent need to develop effective treatment strategies for IS. Our findings revealed the opposing roles of ELP4 and ZEB2 in TCF7L2 expression and microglia M1 polarization during IS development, which provided potential targets for the diagnosis and treatment of IS.

New therapy strategies for regulating the microenvironment to improve functional outcomes and tissue recovery after IS have recently garnered a lot of attention. Microglia are resident immune cells in the CNS, which help maintain normal CNS homeostasis.^27^ Inflammatory microglia express characteristic markers, including CD86, CD16, and CD32, and tend to release destructive mediators such as TNF‐α, iNOS, IL‐1β, and IL‐6, worsening brain injury after IS.^28^ In vivo and in vitro studies demonstrated that inhibiting microglia M1 polarization and promoting microglia M2 polarization protect against IS.^28^, ^29^ TLR4 is a receptor of HMGB1 that is expressed on microglia and mediates neuroinflammatory disorders.^30^ TLR4 can activate NF‐κB signal transduction, and TLR4/NF‐κB is a key signal regulating inflammatory responses.^31^ Notably, the inhibition of TLR4/NF‐κB signal suppressed microglia M1 polarization and neuroinflammation to promote functional recovery during IS development.^32^ Therefore, exploring the regulatory mechanism of TLR4/NF‐κB signal is of great significance for improving microglia M1 polarization and reducing brain injury under IS condition.

TCF7L2 is a risk gene for schizophrenia and autism.^32^ TCF7L2 is a Wnt effector with a particularly prominent role in brain development, including promoting the proliferation of radial glia,^33^ driving oligodendrocyte maturation,^34^ and regulating the terminal selection of thalamic neurons.^35^ TCF7L2 has a function in glutamatergic neuron differentiation and helps maintain neurotransmitter identity.^36^ More importantly, morphine tolerance increased TCF7L2 expression in microglia, TCF7L2 upregulation promoted microglia M1 polarization by activating the TLR4/NF‐κB/NLRP3 pathway.^10^ Our results showed that TCF7L2 silencing reduced MCAO/R‐induced cerebral injury in mice and inhibited OGD/R‐induced inflammatory response and microglia M1 polarization in vitro by inactivating the TLR4/NF‐κB signal. It's suggested that TCF7L2 may be a key factor mediating microglial polarization under IS conditions. Related measures to intervene TCF7L2, such as specific drugs to silence TCF7L2 gene expression and small molecule inhibitors, are expected to be important strategies for the treatment of IS.

ELP4 is a subunit of the elongator complex, and its impairment may be related to several neurological disorders.^13^, ^37^ The role of ELP4 in IS has not been reported before. As reported, ELP4 promoted the transcriptional elongation of target genes by increasing histone acetylation levels of downstream gene promoter.^14^ Interestingly, the similar mechanism was observed in IS. Specifically, ELP4 upregulation promoted microglia M1 polarization and neuroinflammation under IS condition by promoting the enrichment of H3K27ac in the TCF7L2 promoter region and TCF7L2 expression. Prior to this, there have been few reports on ELP4 as an assistant factor to regulate the acetylation level of target factors. Our discovery further deepened the mechanisms of the key factor ELP4 in regulating the development of IS. Of course, other members of the elongator complex also function in regulating the progression of neurological disorders.^12^ Our study did not clarify whether other ELP members are involved in regulating the transcriptional activation of TCF7L2, or whether ELP4 interacts with other histones to jointly regulate the transcriptional activation of TCF7L2, which is worth further exploration.

ZEB2 has an important role in nervous system development.^38^ The role of ZEB2 in IS has been reported. As evidence, ZEB2 upregulation protected neuron from pyroptosis in OGD/R‐treated neurocytes.^39^ In addition, ZEB2‐enriched BMSC‐derived exosomes promoted functional recovery following IS.^18^ Consistently, our results demonstrated that OGD/R markedly inhibited ZEB2 expression in HMC3 cells. As previously reported, ZEB2 achieves its role in diseases by transcriptionally regulating the downstream targets.^40^ However, it was also previously reported that ZEB2 regulated the ubiquitination level of target protein by acting as a ubiquitin E3 ligase.^41^ Of the different types of polyubiquitin chains, K48 and K63 have been found to be the most abundant ubiquitin linkage types in mammalian cells.^42^ The K48‐linked chains serve as proteasomal targeting signals, whereas the K63‐linked chains mainly function in nonproteolytic processes such as inflammatory signaling and endoplasmic reticulum‐mediated endocytosis and degradation.^43^, ^44^ Our findings displayed that ZEB2 enhanced the proteasomal degradation pathway of TCF7L2 by promoting K48‐linked ubiquitination of TCF7L2. However, whether ZEB2 affects other types of ubiquitination degradation pathways of TCF7L2 deserves further exploration in the future.

## 5 CONCLUSION

In summary, our findings showed for the first time that TCF7L2 upregulation exacerbated cerebral injury by promoting microglia M1 polarization under IS condition. Mechanistically, ELP4 and ZEB2 promoted and inhibited TCF7L2 level at transcriptional and post‐translational levels, respectively. Our findings provide novel targets for IS diagnosis and therapy, as well as a theoretical foundation for developing new IS diagnostic and therapeutic methodologies.

## AUTHOR CONTRIBUTIONS

**Xiao‐li Min**: Conceptualization; formal analysis; writing ‐ Original Draft; funding acquisition. **Sixian Lin**: Methodology; validation. **Jia‐yi Hu**: Resources; data Curation. **Rui Jing**: Investigation; visualization. **Qing Zhao**: Resources; supervision. **Fei‐fei Shang**: Validation; project administration. **Yong Zeng**: Writing ‐ Review and Editing; funding acquisition.

## CONSENT FOR PUBLICATION

N/A.

## CONFLICT OF INTEREST STATEMENT

The authors declare that there is no conflicts of interest.

## ETHICS STATEMENT

The animal studies were approved by Ethical Review Committee of Animal Experiments in Kunming Medical University (Ethics Approval No. kmmu20220412).

## Supporting information

Figure S1

## Data Availability

The datasets generated during and/or analyzed during the current study are available from the corresponding author on reasonable request.
