# Supplementary material for: The opposite effect of ELP4 and ZEB2 on TCF7L2‐mediated microglia polarization in ischemic stroke
Source: J Cell Commun Signal. 2025 Jan 16;19(1):e12061. doi: 10.1002/ccs3.12061 (PMC11736883; doi:10.1002/ccs3.12061)
Supplement: Supplementary file 1 — Figure S1 [file CCS3-19-e12061-s001.docx]

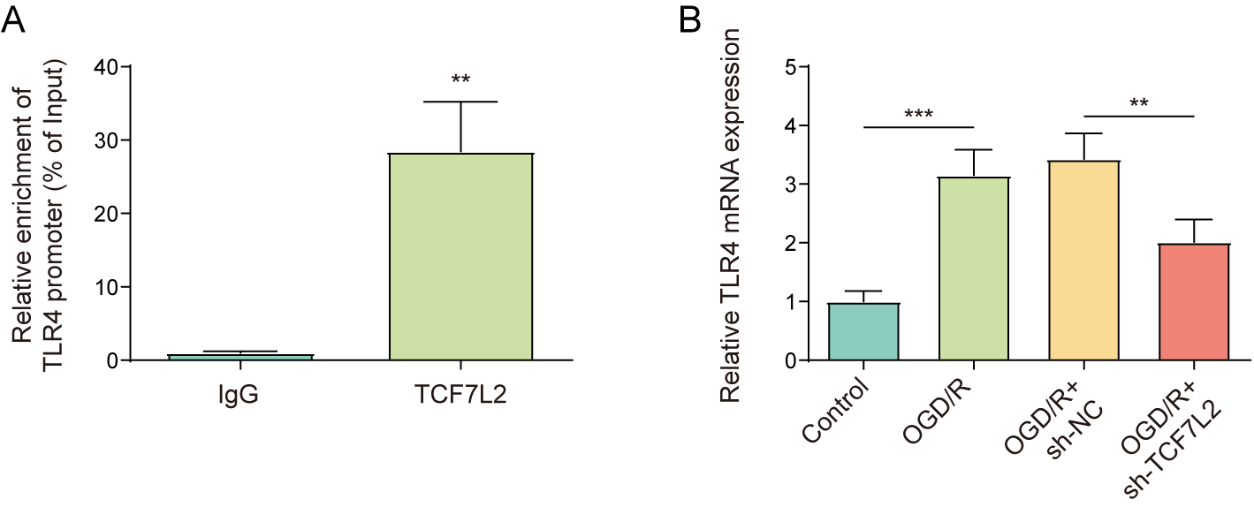
**Supplementary Fig. 1** (A) The binding relationship between TCF7L2 and TLR4 promoter was analyzed by ChIP assay. (B) HMC3 cells were transfected with sh-NC or sh-TCF7L2 combined with OGD/R treatment, and TLR4 mRNA level in cells was detected by RT-qPCR. Data were expressed as mean ± SD. All our data were obtained from three independent experiments. ***p*< 0.01, ****p*< 0.001.
